# High-Pressure Homogenization and Biocontrol Agent as Innovative Approaches Increase Shelf Life and Functionality of Carrot Juice

**DOI:** 10.3390/foods10122998

**Published:** 2021-12-04

**Authors:** Davide Gottardi, Lorenzo Siroli, Giacomo Braschi, Samantha Rossi, Federico Ferioli, Lucia Vannini, Francesca Patrignani, Rosalba Lanciotti

**Affiliations:** 1 Department of Agricultural and Food Sciences, Campus of Food Science, Piazza Goidanich 60, 47521 Cesena, FC, Italy; davide.gottardi2@unibo.it (D.G.); lorenzo.siroli2@unibo.it (L.S.); giacomo.braschi2@unibo.it (G.B.); samantha.rossi@unibo.it (S.R.); federico.ferioli@unibo.it (F.F.); lucia.vannini2@unibo.it (L.V.); francesca.patrignani@unibo.it (F.P.); 2 Interdepartmental Centre for Agri-Food Industrial Research, Campus of Food Science, Via Quinto Bucci 336, 47521 Cesena, FC, Italy

**Keywords:** high-pressure homogenization, shelf life, biocontrol, safety, vegetable drink

## Abstract

Recently, application of high-pressure homogenization (HPH) treatments has been widely studied to improve shelf life and rheological and functional properties of vegetable and fruit juices. Another approach that has drawn the attention of researchers is the use of biocontrol cultures. Nevertheless, no data on their possible combined effect on fruit juices shelf life and functionality have been published yet. In this work, the microbial, organoleptic, and technological stability of extremely perishable carrot juice and its functionality were monitored for 12 and 7 days (stored at 4 and 10 °C, respectively) upon HPH treatment alone or in combination with a fermentation step using the biocontrol agent *L. lactis* LBG2. HPH treatment at 150 MPa for three passes followed by fermentation with *L. lactis* LBG2 extended the microbiological shelf life of the products of at least three and seven days when stored at 10 °C and 4 °C, respectively, compared to untreated or only HPH-treated samples. Moreover, the combined treatments determined a higher stability of pH and color values, and a better retention of β-carotene and lutein throughout the shelf-life period when compared to unfermented samples. Eventually, use of combined HPH and LBG2 resulted in the production of compounds having positive sensory impact on carrot juice.

## 1. Introduction

A fruit- and vegetable-rich diet has a positive impact on human health and wellbeing due to the presence of functional and bioactive compounds, such as phenolic antioxidants, carotenoids, vitamins, and flavonoids [1]. The World Health Organization recommends an introduction of at least 400 g of fruit and vegetables per day in adults [2]. Therefore, their consumption as juices, rather than fresh products, is moving in this direction [3].

The growing consumer interest in healthier food and drinks is projecting that the global juice market will increase in the next years. Among vegetable-based products, carrot juice is one of the most popular non-alcoholic beverages consumed in northern Europe [4]. It is a natural source of antioxidants, such as α- and β-carotene, the precursors of vitamin A and polyacetylene, with anti-tumor properties [4,5,6,7]. Because of its high pH and high sugar content, spoiling and pathogenic microorganisms can easily grow, affecting the shelf life and safety of the product [8,9]. For this reason, fresh carrot juice should be consumed one–two days from production [10]. To extend its shelf life, thermal treatments are commonly applied at the industrial level. However, other than being energetically unsustainable, these treatments may result in undesirable biochemical and nutritional changes, with negative impacts on their sensory properties (for example, pH, taste, and color) [11]. Another possibility is using chemical preservatives, however, this does not always lead to satisfactory results and are not well accepted by consumers [12].

The need for more sustainable approaches has favored the development of non-thermal technologies with the aim to preserve functional and sensorial characteristics of products while guaranteeing their microbial stability [13,14,15,16,17]. Among them, high-pressure homogenization (HPH) can significantly reduce both naturally occurring or intentionally added microorganisms, improving the safety and shelf life of the products [16]. Moreover, it induces physical matrix modifications (changes in pH, viscosity, and particle dimension) [18,19], increasing [20]—or not altering—the presence of functional compounds [21]. This is a very important aspect regarding food differentiation.

Other than physical treatments, addition of natural antimicrobials (essential oils) [22,23] or biocontrol cultures [9,24] have also been proposed to replace chemical preservatives. From an industrial point of view, use of lactic acid bacteria to ferment vegetable and non-dairy beverages is gaining more and more interest [25,26]. In fact, fermentations that apply tailored bacteria represent a fundamental tool to increase safety and shelf life, as well as preserving and increasing the functionality and the sensorial properties of vegetable drinks and juices [25,27,28,29]. Siroli et al. [9] described the potential of three nisin-producing *Lactococcus lactis* strains (LBG2, 3LC39, and FBG1P) as a tool to stabilize fermented carrot juice and soymilk from a microbiological point of view. In particular, strain LBG2, having a rapid fermentation kinetic (pH reduction) and a high nisin production in carrot juice, exerted a strong anti-listeria activity and improved the sensory profile of pre-heated carrot juice. On the other hand, the bacteriocin nisin, the first authorized to be used as a natural food preservative, is a natural antimicrobial with a wide range of actions [30,31]. While numerous studies have shown the antimicrobial properties of nisin deliberately added in vegetable juices, only a few of them have reported the use of biocontrol strains to produce nisin in situ [9].

Since, to our knowledge, there are no studies exploring the combination of these two sustainable approaches, the main objective of this study was to investigate the combined effect of HPH and biocontrol culture (*L. lactis* LBG2) on carrot juice quality and stability. The shelf life of the products obtained was evaluated at two different temperatures of storage (4 and 10 °C). In particular, the growth kinetic of the indigenous microbes (total mesophiles, yeasts, and coliforms) and the viability of LBG2 (when applied) were followed over time during the storage period. Moreover, color, volatile molecule profiles, and carotenoids were measured throughout the storage period of the carrot juice.

## 2. Materials and Methods

### 2.1. Carrot Juice

Carrot juice was prepared using fresh carrots, as described by Siroli et al. [9]. Briefly, carrots were steeped in a solution containing 100 ppm of Sodium hypochlorite for 2 min for sanitization [32]. Then, they were wiped up, sliced, and placed in a domestic extractor (Russel Hobbs). The resulting extract was collected in a sterile flask. All the trials were conducted using three biological replicates (n = 3).

### 2.2. Selection of the Appropriate High-Pressure Homogenization Treatment (HPH)

The carrot juice, prepared as reported above, was immediately subjected to HPH treatment using a PANDA high-pressure homogenizer (GEA, Parma, Italy), able to reach 220 MPa, and provided with a C and a R-type valve and a thermal exchanger. The valve assembly comprised a ceramic ball-type impact head, a stainless steel large inner diameter impact ring, and a carbide passage head made of tungsten. The homogenizer was previously washed with 1% NaOH water solution, hot water, and finally refrigerated sterilized water. The test was carried out by setting two different juice inlet temperatures: 25 and 50 °C.

Two liters of product, at the two different temperatures, were then subjected to different HPH treatments: 0.1 MPa and 150 MPa for 1, 3, and 5 passes. The HPH passes were carried out in the presence of a thermal exchanger to avoid temperature increase caused by homogenization treatment. The samples subjected to the different treatments were then collected in sterile containers and the total microbial load was determined immediately after the treatment. Decimal dilutions were distributed in plate count agar (PCA) plates (Oxoid, Milan, Italy) and colonies were counted after 48 h incubation at 30 °C.

### 2.3. Shelf-Life Assessment of Carrot Juice Considering the Combination of HPH Treatment and Biocontrol Agent

In these trials, non-thermal treatments such as HPH and fermentation with a nisin-producing *Lactococcus lactis* strain (LBG2) were combined to stabilize carrot juice. Samples were divided into three groups: (i) control; (ii) HPH; (iii) HPH plus LBG2. For the carotenoid measurements, pasteurized (72 °C for 15 min) carrot juice was also considered.

#### 2.3.1. HPH Treatment

Two liters of raw organic carrot juice was subjected to HPH treatment at 0.1 MPa (used as control) or 150 MPa for 3 passes (selected based on the previous trial). All the HPH treatments were performed according to the methodology reported above. The inlet temperature of the juice was 25 °C and the HPH passes were carried out in the presence of a thermal exchanger. The controls and treated samples were collected in sterilized glass bottles prior to the shelf-life study.

#### 2.3.2. Fermentation Agent

The biocontrol *L. lactis* LBG2 belonged to the Culture Collection of the Department of Agricultural and Food Sciences, University of Bologna. This strain, isolated from cow milk, is a nisin Z-producer [9]. The strain was also previously characterized for fermentative potential in milk, soymilk, and carrot juice, and for nisin production, antimicrobial activity, and modification of the volatile molecules of milk, soymilk, and carrot juice [9]. The strain was preliminarily grown in M17 broth (Oxoid, Milan, Italy) for 24 h at 30 °C, then refreshed two times in M17 broth for 24 h at 20 °C before the fermentation trials. The proper samples were inoculated with the biocontrol at a concentration of 10^6^ cfu/mL and then left to ferment for 7 h at 30 °C prior to the shelf-life assessment.

### 2.4. Shelf-Life Assessment

All the samples were stored at two different temperatures, 4 and 10 °C, and followed for 12 and 7 days, respectively. According to the temperature applied, aliquots were collected over time (2, 6, 9, and 12 or 1, 2, 5, and 7 days, respectively) for microbiological, sensorial, and chemical analyses.

### 2.5. Microbiological Analyses, pH, Nisin Concentration

Cell loads of yeasts, total coliforms, total mesophiles, and lattococci (or *L. lactis* LBG2) were determined by plate counting on yeast peptone dextrose (YPD) (Oxoid, Milan, Italy), violet red bile lactose agar (VRBA) (Oxoid, Milan, Italy), PCA, and M17, respectively. Decimal dilutions of the samples, performed in Ringer solution [0.9% (*w*/*v*) NaCl], were inoculated in Petri dishes incubated 48 h at 30 °C for YPD, M17, PCA, and 24 h at 37 °C for VRBA. The pH was measured by using a pH-meter Basic 20 (Crison Instruments, Barcelona, Spain).

Nisin activity determination was performed by the agar well diffusion method as described by Siroli et al. [9].

### 2.6. Color Analysis

Color was measured by a Minolta^®^ CR-400 colorimeter (Milan, Italy) using the CIELab scale and Illuminant D65. The instrument was calibrated with a white tile (L* 98.03, a* −0.23, b* 2.05) before the measurements. Results were expressed as L*, a*, and b*. *ΔE* (total color difference) was calculated according to the following Formula:

(1) $$\Delta E=\sqrt{{\left(L^{*}-L_{0}^{*}\right)}^{2}+{\left(a^{*}-a_{0}^{*}\right)}^{2}+{\left(b^{*}-b_{0}^{*}\right)}^{2}}$$

### 2.7. Volatile Molecule Profiles

The volatile molecule profiles were detected with GC/MS/SPME technique, as described by Siroli et al. [9]. Briefly, the samples were analyzed immediately after the treatments and after the different storage periods until reaching the shelf life. A CAR/PDMS, 75 μm fiber (SUPELCO, Bellafonte, PA, USA) was used to perform the solid-phase microextraction (SPME). The samples (5 mL) were placed in vials and incubated for 10 min at 45 °C. Then the fiber was exposed to the vial headspace for 30 min at 45 °C. The volatile molecules adsorbed were desorbed in the gas chromatograph (GC) injector port in splitless mode at 250 °C for 10 min. The headspace of the volatile compounds was analyzed using Gas-Chromatography (GC) 6890N, Network GC System with mass spectrometry (MS) 5970 MSD (Agilent Hewlett–Packard, Geneva, Switzerland). The column used was J & W CP-Wax 52 CB (50 m × 320 μm × 1.2 μm). The initial temperature was 40 °C for 1 min and then was increased by 4.5 °C/min up to 65 °C. After that, the temperature increased by 10 °C/min up to 230 °C and remained at this temperature for 17 min. Compounds were identified by comparison based on a NIST 11 (National Institute of Standards and Technology) database. Gas carrier was helium at 1.0 mL/min flow.

### 2.8. Carotenoid Content

#### 2.8.1. Extraction of Carotenoids from Carrot Juice

Carotenoids were extracted from carrot juice samples according to Purkiewicz et al. [33], with some modifications. Briefly, a volume of 0.5 mL of juice was transferred to a 10-mL Teflon screw cap glass tube with 1.5 mL of n-hexane, 1.5 mL of acetone, and 5 mL of a 10% (*w*/*v*) sodium chloride solution used to avoid the formation of an emulsion. The mixture was then stirred on a vortex stirrer for 10 s and centrifuged at 662× *g* for 2 min. The organic supernatant fraction was transferred to a second tube, and the extraction procedure was repeated four times more on the residual phase with 1.5 mL of n-hexane each time. The pooled organic extracts were washed with 2 mL of water, stirred for 10 s, and then centrifuged at 662× *g* for 2 min. The separated hexane phase was moved to a 100-mL flat bottom flask, dried under reduce pressure in a rotary evaporator (bath temperature: 25 °C), kept under a nitrogen flow for 30 s, dissolved in 3 mL of acetone, transferred after a brief stirring in two 1.5-mL PP centrifuge tube, and kept at −18 °C until HPLC analyses (up to three days). Solvents were of analytical grade and purchased from Merck (Darmstadt, Germany).

#### 2.8.2. Determination of Carotenoids by High-Performance Liquid Chromatography (HPLC)

Analyses were carried out on a HPLC apparatus from Jasco (Tokyo, Japan), equipped with two binary pumps (mod. PU-1580), a diode array UV-VIS detector (mod. MD-1510, quartz flow cell, optical path: 10 mm), and an autosampler (mod. AS-2055 Plus). Data were processed by the software ChromNAV (ver. 1.16.02) from Jasco. The solvent system consisted of two mobile phases: (A) water, (B) acetone; both solvents purchased from Merck were of chromatographic grade, filtered (0.45 μm), and degassed prior their use. The gradient program was the following: 0–5 min, 35% A; 5–9 min, 35 to 10% A; 9–12 min, 10% A; 12–14 min, 10 to 0% A; 14–17 min, 0% A; 17–19 min, 0 to 35% A; 19–30 min, 35% A as post run (total method time: 30 min). The flow rate and the injection volume were 0.8 mL/min and 5 μL, respectively. Chromatograms were acquired at 450 nm, whereas absorption spectra were recorded from 400 to 650 nm. Compound separation was performed by a Kinetex 2.6 μ C18 100A column (75 × 4.6 mm i.d., particle size: 2.6 μm) equipped with a guard cartridge Gemini-NX (4.0 × 3 mm i.d.), both from Phenomenex (Torrance, CA, USA). Colum temperature was maintained at 30 °C throughout analyses. Before HPLC determination, extracts were centrifuged at 15,000× *g* for 3 min at 10 °C and then filtered in HPLC amber glass vials through RC syringe filters (diameter: 13 mm; pore dimension: 0.45 μm) from GVS Filter Technology (Indianapolis, IN, USA). Compound identification was assessed comparing peak retention times with those of a standard compound (β-carotene) and considering the results illustrated by Purkiewicz et al. [33]. External standard mode was applied as a quantification method, constructing a β-carotene calibration curve using a range between 0.00025–0.04909 mg/mL (eight calibration points, *r* > 0.99). Lutein was quantified using β-carotene as a reference compound at the following concentration levels: 0.00025–0.00491 mg/mL (five calibration points, *r* > 0.99). The limit of detection (LOD) and the limit of quantification (LOQ) of the method for β-carotene were 0.00010 and 0.00023 mg/mL of juice, respectively.

### 2.9. Statistical Analysis

Microbial cell loads, color, and volatile profiles were analyzed using the one-way ANOVA option of Statistica software (v. 8.0; StatSoft, Tulsa, OK, USA). The significance of data obtained was evaluated using ANOVA followed by LSD test at *p* < 0.05. The volatile molecule profiles were analyzed using a principal component analysis (PCA) performed by Statistica software (v 8.0; StatSoft, Tulsa, OK, USA).

## 3. Results

### 3.1. Selection of the Appropriate High-Pressure Homogenization Treatment (HPH)

In a preliminary phase, the effectiveness of different hyperbaric treatments against the microflora naturally present in carrot juice was evaluated in order to find the best condition for reducing the naturally occurring microflora. In particular, the antimicrobial effect of HPH treatments at 150 MPa was assessed on carrot juice considering two different inlet temperatures (25 and 50 °C), and three different numbers of HPH passes (one, three, and five). The control was represented by carrot juice subjected to a pressure treatment of 0.1 MPa for a single pass.

Table 1 shows the reduction of total mesophilic counts (TMC) immediately after the different HPH treatments, expressed as Δ log CFU/mL according to the homogenizing treatment applied.

The data obtained showed that the antimicrobial effect of the inlet temperature was limited. In fact, for the same pressures applied the effect of the different inlet temperatures (25 or 50 °C) was significant different only when one pass was applied, while no differences were observed with more passes. As expected, the application of 0.1 MPa (atmospheric pressure, the control) did not reduce the microbial load. In contrast, a reduction of more than a logarithmic cycle was observed after one pass at 150 MPa, despite the inlet temperature used. Increasing the number of passes to 150 MPa resulted in an additive effect on the observed microbial load reduction. In fact, three passes at 150 MPa led to a TMC reduction ranging between 1.95 and 2.08 log CFU/mL, about one logarithmic cycle higher than the cell load reduction observed with a single pass. An increase of the microbial deactivation was also observed with the application of five passes at 150 MPa. However, the increase in microbial load reduction was not significantly different from the three pass treatment. In fact, it ranged between 0.26 and 0.30 logarithmic cycles, regardless of the inlet temperature considered.

Based on these results, three passes of HPH treatment at 150 MPa with an inlet temperature of 25 °C was selected as the optimal one to be combined with the use of *L. lactis* LBG2 for carrot juice stabilization. In fact, a proper reduction of natural microflora is necessary for subsequent LBG2 fermentation to avoid the growth of spoiling microorganisms.

### 3.2. Samples Stored at 4 and 10 °C

The shelf life of the obtained carrot juices, along with their chemical and functional stability, was then studied at 4 and 10 °C to simulate refrigerated and unfavorable storage conditions, respectively. The samples considered were control (not treated carrot juice), HPH (carrot juice subjected to HPH treatment at 150 MPa), and HPH plus LBG2 (carrot juice subjected to HPH treatment at 150 MPa followed by fermentation by *L. lactis* LBG2).

#### 3.2.1. pH

**Figure 1:**
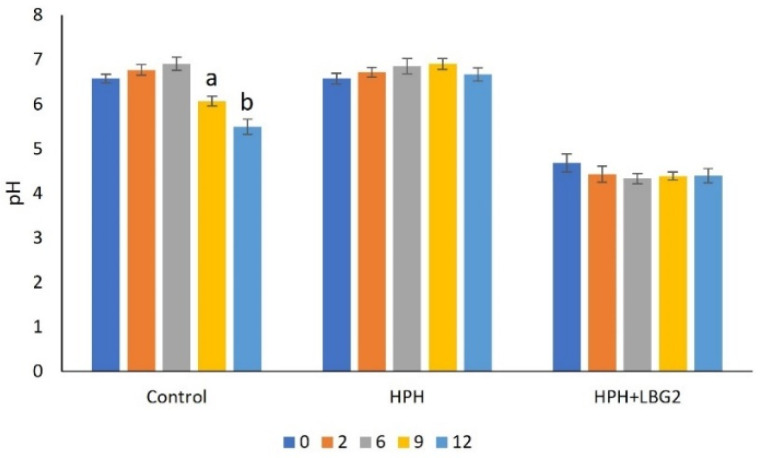
pH of treated and untreated carrot juice stored at 4 °C and followed for 12 days. Control: not treated; HPH: carrot juice subjected to HPH treatment; HPH + LBG2: carrot juice subjected to HPH and then fermented with the biocontrol agent *L. lactis* LBG2. Different letters mean statistically significant differences within a treatment (*p* < 0.05). Results are the mean of 3 biological repetitions (n = 3).

Fresh carrot juice had an initial pH of 6.58, and this value was not significantly modified upon HPH. After HPH, part of the sample was inoculated with LBG2 (initial concentration: 6.0 log CFU/mL) and left to ferment for 7 h, allowing *L. lactis* growth, nisin production, and juice acidification. After fermentation, these samples contained 9.5 log CFU/mL of LBG2, around 13 mg/L of nisin (data not shown), and they had a final pH of 4.68. During storage at 4 °C for 12 days, the pH was measured over time (Figure 1). HPH and HPH + LBG2-treated samples showed a stable pH, with minor fluctuation over time (final pH 6.7 and 4.4, respectively). On the contrary, control samples had a pH that decreased from day six, reaching a value of 5.5 on day 12.

**Figure 2:**
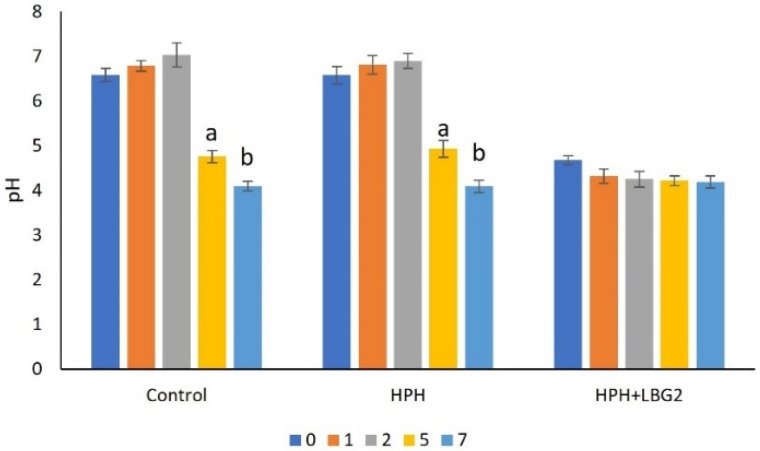
pH of treated and untreated carrot juice stored at 10 °C and followed for 7 days. Control: not treated; HPH: carrot juice subjected to HPH treatment; HPH + LBG2: carrot juice subjected to HPH and then fermented with the biocontrol agent *L. lactis* LBG2. Different letters mean statistically significant differences within a treatment (*p* < 0.05). Results are the mean of 3 biological repetitions (n = 3).

For samples stored at 10 °C, pH was followed for seven days (Figure 2). The low pH (4.7) of the HPH + LBG2 samples remained quite constant over time, reaching pH 4.2 after seven days. On the other hand, the control and HPH-treated sample showed a progressive pH reduction after two days, stabilizing at 4.1 after seven days.

Regarding the nisin concentration, the presence of the bacteriocin was not observed in control and HPH-treated samples (data not shown). In contrast, samples inoculated with LBG2 contained 13 mg/L of nisin after fermentation. This value showed a progressive decrease over time, but it never reached concentration below 4 mg/L, independent from the sampling time and storage temperature applied (data not shown).

#### 3.2.2. Microbiological Analyses

Fresh carrot juice was characterized by an indigenous population of 5.1 log CFU/mL of TMC, 3.4 log CFU/mL coliforms, and 3.8 log CFU/mL of yeasts. The HPH treatment determined an immediate reduction of about 1.6, 1.3, and 2.1 log CFU/mL for TMC, yeasts, and total coliforms, respectively, reducing the initial microbial load as observed during the preliminary tests. The antimicrobial potential of the fermented samples was observed during the storage period. The shelf life of control samples stored at 4 °C was less than six days. In fact, at this time point TCM values exceeded 6 log CFU/mL (Table 2). On the contrary, HPH samples that started with a lower TMC reached 5.6 log CFU/mL only after nine days. The addition of the biocontrol on top of the HPH treatment kept TMC stable below 4.4 log CFU/mL during the entire 12 days. Storage at 10 °C allowed an increase of TCM up to 6.4 and 9.0 log CFU/mL after two and five days, respectively, in control samples. Those subjected to HPH showed similar TMC loads only after five and seven days, respectively. On the other hand, samples treated with HPH and fermented with LBG2 did not show an increase in TMC and remained below 4 log CFU/mL until the end of the trial.

Total coliforms in control samples stored at 4 °C were stable over time (ranging between 3.4 and 4.1 log CFU/mL), while an initial reduction ranging between 1.8 and 2.5 log CFU/mL immediately after HPH was observed in HPH and HPH-LBG2 samples. During the storage at 4 °C total coliforms reached values below the detection limit after six days of storage in all the considered samples (data not shown). In control samples stored at 10 °C, total coliforms remained stable over time (Table 3). However, in this case, although HPH determined a reduction of total coliforms, their concentration increased up to 2.0 log CFU/mL after five days. The fermentation with LBG2 on top of the HPH treatment avoided this increase, maintaining total coliforms below 1 log CFU/mL after two days and maintained this parameter for the whole period of storage.

Yeasts were around 3.8 log CFU/mL in untreated samples and their load increased up to 4.5 log CFU/mL after nine days of storage at 4 °C. Application of HPH treatment reduced yeast counts to 2.5 log CFU/mL. However, only the combination of HPH and LBG2 was able to keep yeasts below 4 log CFU/mL for 12 days when the samples were stored at 4 °C (Table 4). Storage at 10 °C favored the growth of yeasts in untreated samples to 4.4 and 6.1 log CFU/mL on day two and five, respectively. Since the latter value was already above the acceptable limit of yeast in juices, no counts were performed on day seven. On the contrary, yeasts never exceeded the cell load of 4 log CFU/mL in treated samples (both HPH and HPH + LBG2) during the time of storage considered.

Independently from the storage temperature of 4 and 10 °C, the biocontrol agent LBG2 maintained a constant cell load, around 9.0 log CFU/mL, for the whole period of storage (data not shown).

#### 3.2.3. Color Analyses

Lightness (L*) and chromatic parameters (a*, b*) of treated and untreated carrot juices were measured and compared with those of untreated samples (Table 5). Immediately after production, HPH and HPH + LBG2-treated carrot juices showed a reduction of luminosity L* (control 50.6, HPH 46.5, HPH + LBG2 47.9). However, in the fermented product this decrease was not significant. Moreover, both the treated samples showed an increase of chromatic indexes (a* control 14.8, average treated 18.6; b* control 38.8, average treated 45.8). In particular, a* and b* were higher in HPH + LBG2 (20.1 and 47.9, respectively) than HPH alone (17.2 and 43.7, respectively). During storage at 4 °C for 12 days, L* decreased in control samples while it remained stable in HPH and HPH + LBG2 samples. Even a* stayed constant in treated samples while it increased in control. On the contrary, b* values decreased in almost all samples considered. Overall, HPH + LBG2 treatment maintained the three parameters with the highest scores even after 12 days. During the storage at 10 °C, L* and a* were not significantly affected by the storage time, while b* decreased in the treated samples, reaching similar values as the control after seven days. The total color difference (ΔE*ab) values between fresh juice and HPH or HPH plus LBG2, immediately after the treatments, were 6.8 and 5.3 CIELAB units, respectively. However, ΔE*ab increased mainly in control samples over time (7.6 CIELAB units), compared with treated samples (around 4.8 CIELAB units) at the end of storage.

#### 3.2.4. Influence of HPH and HPH Combined with LBG2 on the Stability of Carotenoids and Profile Changes

**Figure 3:**
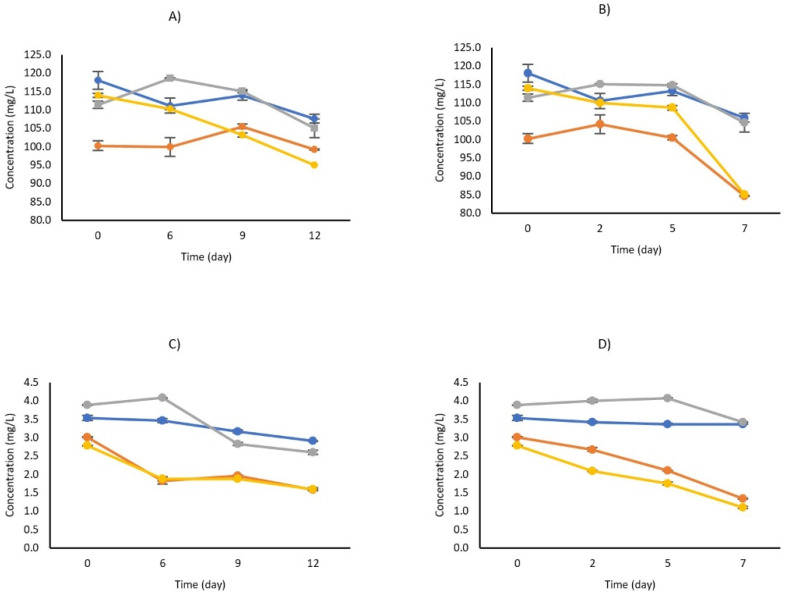
β-carotene (**A**,**B**) and lutein content (**C**,**D**) in carrot beverages over time during storage at 4 (**A**,**C**) or 10 °C (**B**,**D**). Fresh carrot juice (blue) was treated with high-pressure homogenization (HPH) (orange), HPH and subsequent fermentation with LBG2 (gray), thermal treatment (yellow). Control is reported in blue. Results are the mean of 3 biological replicates (n = 3).

Data on the carotenoids measured in the different samples are reported in Figure 3. The total carotenoids quantified (β-carotene + lutein) in fresh carrot juice were 121.5 mg/L of juice, which is in line with the range (30–300 mg/L) specified by AIJN. Untreated carrot juice showed a reduction of β-carotene and lutein over time, mainly after six–seven days, both at 4 and 10 °C. Application of HPH determined an instant reduction of β-carotene, from 118 to 100 mg/L, and lutein, from 3.5 to 3 mg/L. Storage at 4 °C did not further impact the level of β-carotene while it reduced lutein to 1.8 mg/L after six days. Storage at 10 °C for seven days instead reduced β-carotene and lutein to 85 and 1.3 mg/L, respectively. Interestingly, a combination of HPH with LBG2 fermentation showed a positive effect on both β-carotene and lutein concentration. In fact, their initial concentration was similar to that reported for fresh juice. Moreover, their decay profiles were significantly lower than those observed for HPH alone. After the storage period, both at 4 and 10 °C, the final levels of the two compounds were not significantly different from untreated samples. For this carotenoid quantification, a thermal-treated carrot juice was also considered. While the thermal treatment did not impact the initial concentration of β-carotene, it affected the levels of lutein. Their evolution over time at 4 and 10 °C followed the same trend observed for HPH-treated samples, reaching similar values at the end of the storage.

#### 3.2.5. Volatile Molecule Profiles

The analysis of the volatile compounds of carrot juices immediately after their productions allowed us to identify around 85 molecules belonging to different classes of compounds (Table S1), which can provide interesting information about relative changes in aroma composition. Data are expressed as percentage of the peak area of each compound with respect to the total area (which are reported in the tables).

As reported by Siroli et al. [9], the most abundant compounds belonged to terpenes and terpenoids (mainly Caryophyllene, γ-Terpinene, and Terpinolene), esters, and ketones (Table 6). Compared to untreated samples, HPH samples showed a higher abundance of terpenes, terpenoids, and acids (acetic acid) immediately after the treatment. On the other hand, samples that went through the double action of HPH and LBG2 fermentation had a higher relative abundance of alcohols (i.e., Terpinen-4-ol) and ketones (diacetyl, 2,6-dimethyl-4-heptanone). Interesting, the relative abundance of myristicin was lower in samples when HPH and LBG2 were combined (Table S1).

The volatile molecule profiles of the samples stored at 4 and 10 °C were determined only on samples collected within their shelf-life period, as described in the previous paragraph.

Compared with the original product, the storage of the control samples at 4 °C within six days (shelf-life period) showed an increase of the relative abundance of aldehydes, alcohols, and terpenes, with a decrease in ketones. HPH-treated juices were characterized by a higher abundance of alcohols, and the same modification was observed for HPH + LBG2-treated samples where they were accompanied by an increase in terpenes and aldehydes with a reduction in ketones. This trend was also observed during the following days of storage for the treated samples. Even the storage of the control samples at 10 °C for two days (shelf-life period) showed an increase of the relative abundance of alcohols and terpenes, while HPH samples were characterized by a higher abundance of ketones. On the other hand, HPH + LBG2 samples had a higher abundance of terpenes and alcohols with a reduction in ketones. The main group of compounds remained similar during the storage except ketones decreased over time in both treated samples.

**Figure 4:**
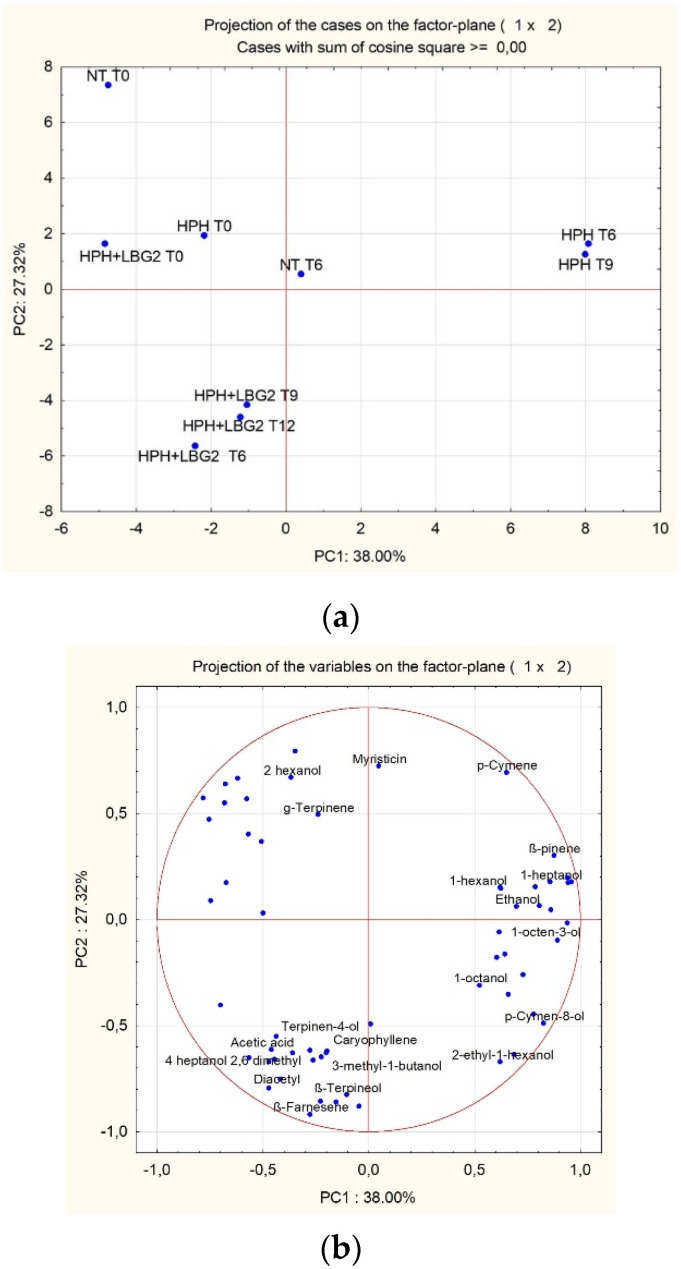
Projection on the factor plane (1 × 2) of carrot juice samples (**a**) and the variables (**b**) when stored at 12 °C up to the sample shelf life (NT, 6 days; HPH, 9 days; HPH + LBG2, 12 days). NT is the control not treated; HPH: carrot juice subjected to HPH treatment; HPH + LBG2: carrot juice subjected to HPH and then fermented with the biocontrol agent *L. lactis* LBG2. For better understanding, only the variables discussed in the text were kept in (**b**).

To better highlight the effects of the different treatments, GC/MS/SPME data, immediately after production and after storage at 4 °C, were analyzed by principal component analysis (PCA). The projection of the samples is reported in Figure 4a where PC1 and PC2 can explain around the 65% of the total variance among the samples. The first cluster included all the samples immediately after production (T0). The second cluster was represented by HPH-treated samples at six and nine days of storage. In group 3, all the HPH + LBG2 samples clustered together, showing no major differences within the 12 days of shelf life. Figure 4b shows the molecules responsible for the cluster of the samples. Among the molecules characterizing HPH + LBG2 there are diacetyl, β-Terpineol, β-farnesene, and Terpinen-4-ol, while HPH-treated samples were mainly characterized by ρ-cymene, 1-octen-3-ol, β-pinene. Moreover, it was confirmed that the sequential action of HPH and LBG2 determined a constant lower abundance of myristicin. Eventually, control samples were characterized by a higher relative abundance of ethanol after six days of storage. This increase was also observed in HPH-treated samples but only after nine days, in line with the growth of possible spoilage yeasts, as reported above. On the contrary, during the 12 days of storage no increase in ethanol abundance was observed in HPH + LBG2 samples.

**Figure 5:**
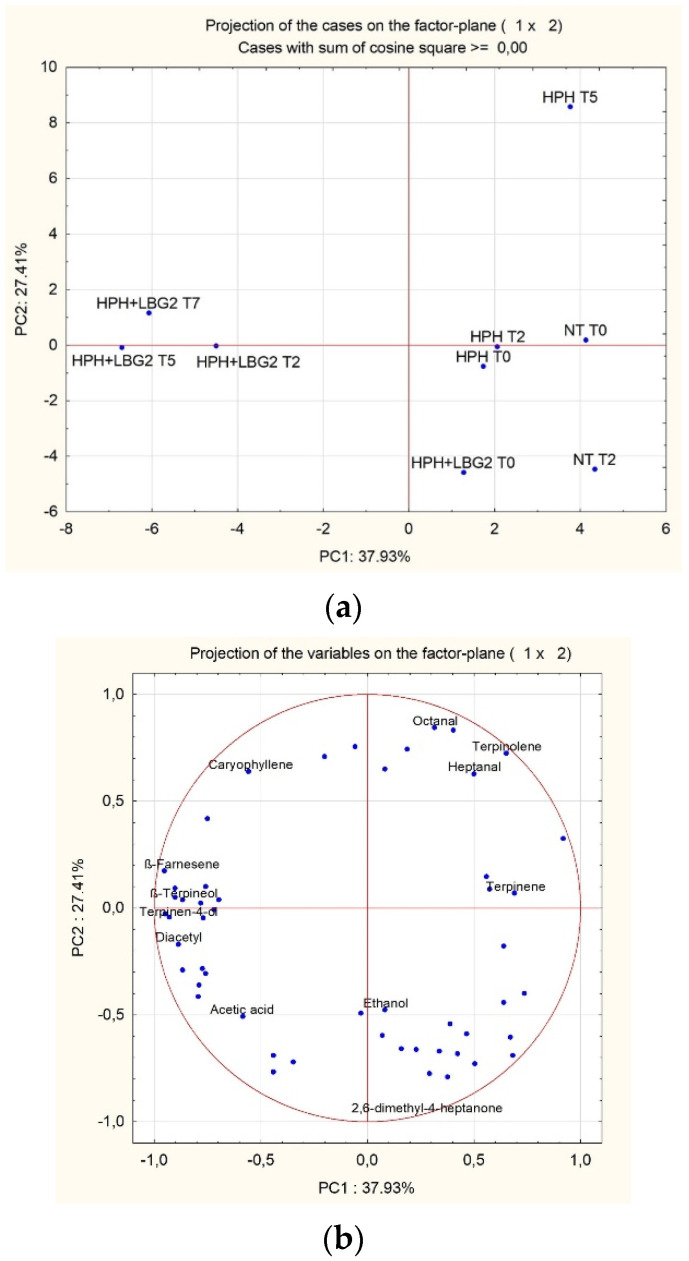
Projection on the factor plane (1 × 2) of carrot juice samples (**a**) and their variables (**b**) when stored at 10 °C up to the sample shelf life (NT, 2 days; HPH, 5 days; HPH + LBG2, 7 days). NT is the control: not treated; HPH: carrot juice subjected to HPH treatment; HPH + LBG2: carrot juice subjected to HPH and then fermented with the biocontrol agent *L. lactis* LBG2. For better understanding, only the variables discussed in the text were kept in (**b**).

The same analysis was also performed with samples stored at 10 °C (Figure 5a). PC1 and PC2 can explain around the 65% of the total variance among the samples. The first cluster included all the samples immediately after production (T0) and the samples at T2 except HPH + LBG2. The second cluster was represented by HPH + LBG2 samples at day two, five, and seven. Figure 5b shows the molecules responsible for the cluster of the samples stored at 10 °C. Diacetyl, β-Terpineol, β-farnesene, and Terpinen-4-ol were again some of the molecules characterizing HPH + LBG2 samples over time, while ethanol and aldehydes (octanal and heptanal) characterized untreated and HPH-treated samples, respectively.

## 4. Discussion

Over the past years, application of the non-thermal treatment HPH has been studied to improve shelf life and organoleptic and functional properties of vegetable and fruit juices [8,19,22,34,35]. Another approach that has drawn the attention of researchers is the use of natural antimicrobials (such as essential oils or bacteriocins) [22,23] or biocontrol cultures [9,24]. Nevertheless, no data on their possible combined effect on fruit juice shelf life and functionality have been published yet. In this work, the microbial stability of extremely perishable carrot juice and its functionality were monitored for 12 and 7 days (stored at 4 and 10 °C, respectively) upon HPH treatment alone or in combination with a fermentation step with the biocontrol agent *L. lactis* LBG2. 

As already demonstrated in many publications [17,34,35,36], HPH treatment at 150 MPa reduces the naturally occurring microflora present in vegetable juice. In this study, a reduction ranging between 1.0 and 2.4 log cycles was observed for TMC depending on the number of HPH passes and inlet temperature of the treated juice. For instance, Patrignani et al. [16] showed a decrease of yeast of about 2.0 log CFU/mL in kiwi juice following an HPH treatment at 200 MPa for two passes. Moreover, Patrignani et al. [22] showed a reduction of three log cycles following an HPH treatment at 200 MPa × two passes on apple juice deliberately inoculated with *Saccharomyces cerevisiae* at a level of 4.8 log CFU/mL. The HPH potential to inactivate microorganisms depends on both internal (chemical-physical characteristics of the matrix and microbial sensitivity) and external factors (HPH operational procedure) [17,37,38]. Among the external factors, pressure degree and number of passes play an important role as much as the temperature generated during the dynamic pressure applied. In fact, it is estimated that the sample is subjected to an increase of around 2 °C/10 MPa during homogenization. Although for short treatment periods temperature increases were not observed [39,40], in the present work a thermal exchanger was applied to maintain temperature at 25 °C. For what concerns the number of passes through HPH at 150 MPa, the data obtained showed an additive antimicrobial effect when increasing the number of HPH passes applied but without linearity in terms of reduction of microbial load. Literature data concerning the additive effect of the number of HPH passes on microbial deactivation are contradictory. Some authors report a limited microbial deactivation following multiple HPH passes and have attributed this trend to the physiological diversity of microbial populations and the presence of resistant cells from the original microbiota of the matrix able to survive at high pressures [41].

The HPH treatment with the selected parameters (150 MPa × three passes) determined an average reduction of about 1.6, 1.3, and 2.0 log CFU/mL for TMC, yeasts, and total coliforms, respectively. This initial reduction extended the shelf life of carrot juice. In fact, the spoilage threshold limit of TMC and yeasts in vegetable juices is usually considered to be 6.0 log CFU/mL [42,43]. These limits were exceeded in HPH-treated samples only for TMC after nine and five days when stored at 4 and 10 °C, respectively, while controls exceeded the limit after six and two days at 4 and 10 °C, respectively.

For what concerns color parameters, HPH increased a* and b* values while it decreased the L* value. The overall ΔE was 6.8 CIELAB units, in line with what was reported by Szczepańska et al. [34], considering the high variability observed in our study. The effect of HPH on juice color seems strongly dependent on food matrices and treatments. In fact, Calligarsi et al. [44] reported an increase in L* and b* values as well as a decrease in a* values upon HPH treatment on banana juice. Zhou et al. [19] observed a luminosity loss and an increase of red color intensity in mango juice after HPH, while Tribst et al. [45] reported a decrease of L* and a* values of mango nectar after HPH. Despite the initial variation due to HPH treatment which may be determined by oxidative process, color variations were less significant than those observed for the control over time. In fact, at the end of the storage period, HPH samples had higher L*, a*, and b* values than controls. According to literature data, HPH treatment does not impact the level of total carotenoids, especially at pressures ranging from 20 to 150 MPa [46]. Even Szczepańska et al. [36] reported that the level of total carotenoids can only be increased by applying 150 MPa, or higher pressures, for longer time (four passes or more). However, they also observed a different behavior depending on the type of carotenoid. For instance, 150 MPa with four passes increased β-carotene concentration but reduced lutein. In our work, 150 MPa for three passes reduced the concentration of β-carotene and lutein with respect to the untreated samples. Although starting from a lower concentration, storage at 4 °C did not further reduce the total carotenoids measured during the 12 days considered. On the other hand, storage at 10 °C promoted a degradation process from day two until day seven, following a first-order reaction [46]. However, the sum of β-carotene and lutein, at the beginning and at the end of the storage, was still within the range of 30 to 300 mg/L given by the AlJN Code of Practice as a reference value for carrot juice and purees [47].

To boost HPH effects and extend carrot juice shelf life, a fermentation step was performed upon HPH treatment using the biocontrol agent *L. lactis* LBG2. The selection of the biocontrol agent applied in this work was based on the results reported by Siroli et al. [9] that showed the good acidifying and nisin-producing capacity of *L. lactis* LBG2 on the same substrate. However, in this work the fermentation process by LBG2 strain was optimized by reducing fermentation times from 24 to 7 h. In fact, samples treated with HPH and fermented by LBG2 showed a drop in pH to 4.6 and an increase of the biocontrol agent up to 9.0 log CFU/mL after 7 h of fermentation. The rapid fermentation kinetic represents an important feature applicable at an industrial level to reduce energy and working costs [48]. Moreover, lowering the pH, production of organic acids, and bacteriocins can prevent possible contamination by undesirable microorganisms, such as spoilage and pathogens [49]. In this regard, the implementation of the fermentation step with LBG2 did not change the microbial profile already observed immediately after HPH treatment but it exerted an important effect on the microbial stability of the juices during their storage. In fact, using the combined treatments, microbiological shelf life of carrot juice was extended to more than 12 and 7 days when stored at 4 and 10 °C, respectively. In fact, the acceptance threshold for TMC and yeasts, reported as 6.0 log CFU/mL for vegetable juices [42,43], have never been overcome during storage, either at 4 or 10 °C. The observed antimicrobial effect is also correlated to nisin production by LBG2. In fact, at the end of the fermentation process, the presence of 13 mg/L of nisin in fermented carrot juice was determined. The presence of nisin was also detected during the storage at 4 and 10 °C, however a decrease of its concentration was observed over time. According to literature data, nisin production occurs mainly in the late exponential growth phase and the beginning of the stationary phase [9]. Then the physical and compositional characteristics of the substrate may induce modifications of the activity and stability of nisin that can also be degraded by proteases [50].

Initial color modifications were more dependent on the HPH treatment; in fact, even the total color difference (5.3 CIELAB units) was in line with what was reported by Szczepańska et al. [36] for carrot juices treated with 150 MPa for three passes. However, the fermentation step and subsequent acidification of the product did not significantly impact the L* parameter while it maintained the three values (L*, a* and b*) during the storage time, particularly at 4 °C. In fact, samples that underwent the combined treatment had the highest L*, a*, and b* values among the samples considered. The fermentation step had a positive effect also on β-carotene and lutein. In fact, the initial concentration of β-carotene was higher in fermented samples than in those treated with only HPH. Although a reduction (6–7 mg/L) was observed at the end of the shelf life, its concentration remained the highest. A similar profile was also observed for lutein. Demir et al. [51,52] reported that acidified carrot juice, especially with lactic acid, significantly increased the β-carotene content and preserved its stability over time. In fact, acidification can help to release bound carotenoids by making them easily extractable during juice preparation. Moreover, low pH may inhibit the oxidative process by protecting compounds such as β-carotene.

Analyses of the volatile compounds detected in all the samples showed that treatments with HPH or HPH and LBG2 had an impact on the final profiles. HPH itself determined a profile where the relative abundance of terpenes and terpenoids was higher than in control and fermented samples. Modifications in the relative abundance of compounds upon HPH treatment were also described by Patrignani et al. [23], who reported a reduction of the percentage of aliphatic aldehydes and an increase in benzaldehyde and terpineol in apricot juice. As already reported by Siroli et al. [9], samples fermented by the biocontrol agent LBG2 are characterized by volatile molecules deriving from *L. lactis* fermentation. In fact, the latter samples were characterized by a higher abundance of ketones (diacetyl, 3,4-dimethyl-2-pentanone, 2,6-dimethyl-4-heptanone), alcohols (1-octanol, 3-methyl-1-butanol, Terpinen-4-ol), and acids (acetic acid) that last during all the storage periods. These volatile molecules have been previously associated with a positive sensory impact in different fermented juices [53,54,55]. Moreover, as observed by Fukuda et al. [56] and Siroli et al. [9], the microbial detoxification of initially present terpene molecules determined a reduction of their abundance. Similarly, myristicin represents an anti-nutritional compound naturally present in carrots [57]. A significant reduction of its abundance in samples fermented with LBG2 represents an interesting tool that can be used to enhance the nutritional properties of the fermented carrot juice. In fact, it has been already reported that lactic acid fermentation can act as a food detoxification process against anti-nutritional factors such as phytates, saponins, tannins, cyanogens, or trypsin inhibitors [58]. PCA analyses of the volatile compounds showed that samples treated with HPH and the biocontrol agent were different with respect to control and HPH-treated samples, however, they did not change significantly over time, showing the stability of the volatilome during the storage period.

## 5. Conclusions

The results obtained in this study showed that HPH treatment followed by fermentation with the biocontrol agent *L. lactis* LBG2 extended the shelf life of carrot juice by at least three and seven days when stored at 10 °C and 4 °C, respectively, compared to untreated juice. Shelf-life tests under thermal abuse at 10 °C highlighted that samples treated with HPH combined with fermentation were the only ones that did not exceed the spoilage limits for the TMC and yeast during all the times of storage considered. In addition, fermented samples showed higher stability in pH and color values throughout the shelf life compared to unfermented samples. Moreover, the combined treatment improved the functionality of the juice to better retain β-carotene and lutein during storage. LBG2 fermentation produced compounds that had a positive sensory impact on the final products.

Although further sensorial trials for consumer acceptability and scaling up steps are required to transfer this technology into an industrial environment, the data obtained in this work demonstrated that the combination of HPH treatment at 150 MPa × three passes followed by fermentation with the biocontrol agent *L. lactis* LBG2 represents a promising tool to extend the shelf life of carrot juice without any detrimental impact on the characteristics of the product.

## Figures and Tables

**Table 1:** Reduction of the total mesophilic count (TMC) expressed as Δ log CFU/mL based on the different HPH treatment applied (pressure, number of passes, inlet temperature). The initial load of TMC was 5.60 log CFU/mL. Results are the mean of 3 biological repetitions (n = 3). Different letters mean statistically different values (*p* < 0.05).

			TMC Post Treat.
Pressure (MPa)	N° Passes	Inlet T° (°C)	Δ log CFU/mL
0.1	1	25	0.03 ± 0.29 ^a^
150	1	25	1.04 ± 0.29 ^b^
150	3	25	1.95 ± 0.07 ^c^
150	5	25	2.21 ± 0.77 ^c^
0.1	1	50	0.50 ± 0.15 ^a^
150	1	50	1.67 ± 0.16 ^c^
150	3	50	2.08 ± 0.64 ^c^
150	5	50	2.38 ± 0.85 ^c^

**Table 2:** Total mesophilic counts (TMC) (log cfu/mL) on treated (HPH and HPH + LBG2) and untreated (Control) carrot juice samples stored at 4 °C for 12 days and 10 °C for 7 days. Different letters mean statistically significant differences within the same time point (*p* < 0.05). Results are the mean of 3 biological repetitions (n = 3).

	Storage at 4 °C					Storage at 10 °C				
	0	2	6	9	12	0	1	2	5	7
Control	5.1 ± 0.2 ^a^	4.6 ± 0.2 ^a^	6.7 ± 0.4 ^a^	7.7 ± 0.2 ^a^	-	5.1 ± 0.2 ^a^	5.7 ± 0.4 ^a^	6.3 ± 0.2 ^a^	9.0 ± 0.2 ^a^	-
HPH	3.6 ± 0.2 ^b^	3.9 ± 0.1 ^b^	3.5 ± 0.1 ^b^	5.6 ± 0.3 ^b^	7.2 ± 0.2 ^a^	3.6 ± 0.2 ^b^	3.9 ± 0.2 ^b^	4.7 ± 0.2 ^b^	6.4 ± 0.8 ^b^	9.1 ± 0.5 ^a^
HPH + LBG2	3.5 ± 0.1 ^b^	3.7 ± 0.1 ^b^	3.8 ± 0.0 ^b^	3.7 ± 0.1 ^c^	4.4 ± 0.2 ^b^	3.5 ± 0.1 ^b^	3.9 ± 0.2 ^b^	3.7 ± 0.2 ^c^	3.8 ± 0.4 ^c^	3.9 ± 0.1 ^b^

**Table 3:** Total coliforms (log CFU/mL) on treated (HPH and HPH + LBG2) and untreated (Control) carrot juice samples stored at 10 °C for 7 days. Different letters mean statistically significant differences within the same time point (*p* < 0.05). Results are the mean of 3 biological repetitions (n = 3).

	Storage at 10 °C				
	0	1	2	5	7
Control	3.4 ± 0.1 ^a^	4.1 ± 0.1 ^a^	3.4 ± 0.1 ^a^	3.7 ± 0.1 ^a^	-
HPH	0.9 ± 0.1 ^c^	1.8 ± 0.2 ^b^	1.9 ± 0.1 ^b^	2.0 ± 0.1 ^b^	1.9 ± 0.2 ^a^
HPH + LBG2	1.6 ± 0.1 ^b^	0.9 ± 0.2 ^c^	0.8 ± 0.1 ^c^	0.9 ± 0.1 ^c^	1.1 ± 0.1 ^b^

**Table 4:** Yeasts (log CFU/mL) on treated (HPH and HPH + LBG2) and untreated (Control) carrot juice samples stored at 4 and 10 °C for 12 and 7 days, respectively. Different letters mean statistically significant differences within the same time point (*p* < 0.05). Results are the mean of 3 biological repetitions (n = 3).

	Storage at 4 °C					Storage at 10 °C				
	0	2	6	9	12	0	1	2	5	7
Control	3.8 ± 0.3 ^a^	4.4 ± 0.2 ^a^	4.3 ± 0.5 ^a^	4.5 ± 0.1 ^a^	-	3.8 ± 0.3 ^a^	4.0 ± 0.1 ^a^	4.4 ± 0.2 ^a^	6.1 ± 0.1 ^a^	-
HPH	2.5 ± 0.2 ^b^	3.8 ± 0.2 ^b^	2.9 ± 0.2 ^b^	4.1 ± 0.2 ^ab^	4.5 ± 0.2 ^a^	2.5 ± 0.2 ^b^	3.0 ± 0.2 ^b^	3.7 ± 0.2 ^b^	3.9 ± 0.3 ^b^	3.5 ± 0.2 ^a^
HPH + LBG2	2.5 ± 0.2 ^b^	3.1 ± 0.1 ^c^	3.3 ± 0.1 ^b^	3.8 ± 0.1 ^b^	3.9 ± 0.1 ^b^	2.5 ± 0.2 ^b^	2.9 ± 0.2 ^b^	3.5 ± 0.1 ^b^	3.7 ± 0.2 ^b^	3.6 ± 0.2 ^a^

**Table 5:** Lightness (L*) and chromatic parameters (a*, b*) measured in treated and untreated carrot juice using a tristimulus colorimeter. All the samples were stored at 4 or 10 °C and followed over time for 12 and 7 days, respectively. Control: not treated; HPH: carrot juice subjected to HPH treatment; HPH + LBG2: carrot juice subjected to HPH and then fermented with the biocontrol agent *L. lactis* LBG2. Different letters mean statistically significant differences within the same time point (*p* < 0.05). If no letters are added, no significant differences within a time point were observed. Results are the mean of 3 biological repetitions (n = 3).

		Days of Storage at 4 °C					Days of Storage at 10 °C				
		0	2	6	9	12	0	1	2	5	7
L*	Control	50.6 ± 2.5 ^a^	48.1 ± 2.3 ^ab^	49.8 ± 0.5 ^a^	49.0 ± 1.6 ^a^	45.5 ± 0.2 ^a^	50.5 ± 2.5 ^a^	50.3 ± 1.3	49.6 ± 3.0 ^ab^	49.5 ± 0.1 ^a^	-
	HPH	46.5 ± 0.8 ^b^	46.7 ± 0.6 ^a^	46.6 ± 2.2 ^b^	45.5 ± 0.3 ^b^	45.1 ± 0.3 ^a^	46.5 ± 0.8 ^b^	48.7 ± 0.6	47.9 ± 2.1 ^a^	44.8 ± 0.1 ^b^	47.7 ± 2.4
	HPH + LBG2	47.9 ± 0.6 ^ab^	49.6 ± 0.5 ^b^	48.7 ± 0.5 ^ab^	47.8 ± 0.2 ^a^	49.4 ± 0.7 ^b^	47.9 ± 0.6 ^ab^	49.3 ± 1.2	51.5 ± 0.1 ^b^	44.7 ± 0.1 ^b^	45.9 ± 1.0
a*	Control	14.8 ± 3.2 ^a^	16.7 ± 2.3	16.8 ± 0.4 ^a^	17.3 ± 1.2 ^a^	17.1 ± 0.5 ^a^	14.8 ± 3.2 ^a^	15.6 ± 1.4 ^a^	15.6 ± 2.4	16.7 ± 2.1 ^a^	-
	HPH	17.2 ± 0.7 ^b^	16.7 ± 0.2	15.3 ± 1.9 ^a^	16.8 ± 0.1 ^a^	16.1 ± 0.4 ^b^	17.2 ± 0.7 ^b^	13.9 ± 1.0 ^a^	14.7 ± 2.6	17.9 ± 0.1 ^a^	18.4 ± 0.2 ^a^
	HPH + LBG2	20.1 ± 0.4 ^c^	17.7 ± 0.8	21.3 ± 0.4 ^b^	21.9 ± 0.1 ^b^	21.2 ± 1.0 ^c^	20.1 ± 0.4 ^c^	18.6 ± 2.6 ^b^	13.5 ± 0.1	22.7 ± 0.1^b^	21.6 ± 0.5 ^b^
b*	Control	38.8 ± 3.0 ^a^	37.3 ± 1.1 ^a^	39.2 ± 0.1 ^a^	37.2 ± 1.0 ^a^	33.7 ± 1.1 ^a^	38.8 ± 3.0 ^a^	38.9 ± 1.3	38.9 ± 1.2 ^a^	39.2 ± 2.1 ^a^	-
	HPH	43.7 ± 2.4 ^b^	42.5 ± 0.9 ^b^	40.7 ± 0.6 ^b^	40.0 ± 0.6 ^b^	39.2 ± 0.5 ^b^	43.7 ± 2.4 ^b^	41.8 ± 1.4	41.6 ± 2.7 ^a^	41.9 ± 0.1 ^a^	38.8 ± 3.4
	HPH + LBG2	47.9 ± 0.9 ^c^	43.2 ± 1.4 ^b^	43.4 ± 0.7 ^c^	43.1 ± 0.5 ^c^	43.5 ± 0.6 ^c^	47.9 ± 0.9 ^c^	43.1 ± 3.4	34.9 ± 0.0 ^b^	43.1 ± 0.1 ^b^	40.9 ± 0.5

**Table 6:** GC/MS/SPME profiles (expressed as relative abundance) of carrot juices immediately after production and during their storage at 4 and 10 °C. Samples were treated with high pressure homogenization (HPH, 150 MPa × 3 passes) or HPH combined with *L. lactis* LBG2 (L) fermentation. For a control (CTRL), a sample that passed through 0.1 MPa was used. Analyses were performed only on samples collected within their shelf-life period (NT, 6 days; HPH, 9 days; HPH + LBG2, 12 days for samples stored at 4 °C or NT, 2 days; HPH, 5 days; HPH + LBG2, 7 days, for samples stored at 10 °C). Results are the mean of 3 biological repetitions (n = 3).

				Storage at 4 °C						Storage at 10 °C					
Time (Days)	0			6			9		12	2			5		7
Molecules	CTRL 1	HPH	HPH + LBG2	CTRL 1	HPH	HPH + LBG2	HPH	HPH + LBG2	HPH + LBG2	CTRL	HPH	HPH + LBG2	HPH	HPH + LBG2	HPH + LBG2
Aldehydes	0.4	1.0	0.9	0.9	0.9	1.7	1.0	1.5	1.3	0.8	0.7	1.3	0.8	1.4	1.7
Ketones	11.0	5.5	14.8	5.4	2.0	6.1	2.4	3.8	4.8	11.0	6.8	6.6	0.8	4.9	2.9
Alcohols	0.7	0.7	2.1	2.3	2.6	4.1	1.9	4.9	5.0	1.7	0.6	4.3	0.9	4.1	5.5
Acids	0.0	0.3	0.4	0.0	0.2	0.4	0.2	0.3	0.3	0.1	0.1	0.3	0.0	0.2	0.4
Esters	9.8	5.3	5.5	7.3	4.3	4.2	4.4	4.5	4.9	7.6	4.1	5.1	5.0	4.9	4.7
Terpenes and Terpenoids	73.3	84.5	71.7	79.4	84.7	80.6	85.0	81.4	79.5	75.0	84.9	74.4	89.6	80.6	80.9
Others	2.4	1.3	1.0	1.8	1.6	1.1	1.7	1.1	1.2	1.9	1.0	1.1	1.1	1.1	1.3
Total area ^3^	17,500	32,800	17,600	21,400	22,500	28,000	21,700	26,300	23,000	18,600	26,800	22,400	18,900	22,600	24,400

Data are the mean of three different samples. The variability coefficient ranged between 5% and 7%. (1) Sample treated at 0.1 MPa. (2) Values equal to 0 are under detection limit. ^3^. Arbitrary units (×100,000).

## Supplementary Materials

The following are available online at https://www.mdpi.com/article/10.3390/foods10122998/s1, Table S1: GC/MS/SPME complete profiles of treated and untreated Carrot juices during their storage at 4 and 10 °C.

## References

[1] Shahbaz H.M., Kim J.U., Kim S.-H., Park J. (2018). Advances in nonthermal processing technologies for enhanced microbiological safety and quality of fresh fruit and juice products. Food Processing for Increased Quality and Consumption.

[2] World Health Organization (2003). Diet, Nutrition, and the Prevention of Chronic Diseases: Report of a Joint WHO/FAO Expert Consultation.

[3] Wootton-Beard P.C., Ryan L. (2011). Improving public health?: The role of antioxidant-rich fruit and vegetable beverages. Food Res. Int..

[4] Sharma K.D., Karki S., Thakur N.S., Attri S. (2012). Chemical composition, functional properties and processing of carrot—A review. J. Food Sci. Technol..

[5] Pferschy-Wenzig E.-M., Getzinger V., Kunert O., Woelkart K., Zahrl J., Bauer R. (2009). Determination of falcarinol in carrot (*Daucus carota* L.) genotypes using liquid chromatography/mass spectrometry. Food Chem..

[6] Zhang Y., Liu X., Wang Y., Zhao F., Sun Z., Liao X. (2016). Quality comparison of carrot juices processed by high-pressure processing and high-temperature short-time processing. Innov. Food Sci. Emerg. Technol..

[7] Nadeem M., Ubaid N., Qureshi T.M., Munir M., Mehmood A. (2018). Effect of ultrasound and chemical treatment on total phenol, flavonoids and antioxidant properties on carrot-grape juice blend during storage. Ultrason. Sonochemistry.

[8] Patrignani F., Vannini L., Kamdem S.L.S., Lanciotti R., Guerzoni M.E. (2009). Effect of high pressure homogenization on Saccharomyces cerevisiae inactivation and physico-chemical features in apricot and carrot juices. Int. J. Food Microbiol..

[9] Siroli L., Camprini L., Pisano M.B., Patrignani F., Lanciotti R. (2019). Volatile molecule profiles and anti-Listeria monocytogenes activity of nisin producers Lactococcus lactis strains in vegetable drinks. Front. Microbiol..

[10] Kaddumukasa P.P., Imathiu S.M., Mathara J.M., Nakavuma J.L. (2017). Influence of physicochemical parameters on storage stability: Microbiological quality of fresh unpasteurized fruit juices. Food Sci. Nutr..

[11] Barzee T.J., El-Mashad H.M., Zhang R., Pan Z. (2019). Carrots. Integrated Processing Technologies for Food and Agricultural By-Products.

[12] Bearth A., Cousin M.-E., Siegrist M. (2014). The consumer’s perception of artificial food additives: Influences on acceptance, risk and benefit perceptions. Food Qual. Prefer..

[13] Jiménez-Sánchez C., Lozano-Sánchez J., Segura-Carretero A., Fernández-Gutiérrez A. (2017). Alternatives to conventional thermal treatments in fruit-juice processing. Part 1: Techniques and applications. Crit. Rev. Food Sci. Nutr..

[14] Roobab U., Aadil R.M., Madni G.M., Bekhit A.E.D. (2018). The impact of nonthermal technologies on the microbiological quality of juices: A review. Compr. Rev. Food Sci. Food Saf..

[15] Bello E.F.T., Martínez G.G., Ceberio B.F.K., Rodrigo D., López A.M. (2014). High pressure treatment in foods. Foods.

[16] Patrignani F., Mannozzi C., Tappi S., Tylewicz U., Pasini F., Castellone V., Riciputi Y., Rocculi P., Romani S., Caboni M.F. (2019). (Ultra) High pressure homogenization potential on the shelf-life and functionality of kiwifruit juice. Front. Microbiol..

[17] Patrignani F., Lanciotti R. (2016). Applications of High and Ultra High Pressure Homogenization for Food Safety. Front. Microbiol..

[18] Salehi F. (2020). Physico-chemical and rheological properties of fruit and vegetable juices as affected by high pressure homogenization: A review. Int. J. Food Prop..

[19] Zhou L., Guan Y., Bi J., Liu X., Yi J., Chen Q., Wu X., Zhou M. (2017). Change of the rheological properties of mango juice by high pressure homogenization. LWT-Food Sci. Technol..

[20] Sentandreu E., Stinco C.M., Vicario I.M., Mapelli-Brahm P., Navarro J.L., Meléndez-Martínez A.J. (2020). High-pressure homogenization as compared to pasteurization as a sustainable approach to obtain mandarin juices with improved bioaccessibility of carotenoids and flavonoids. J. Clean. Prod..

[21] Wellala C.K.D., Bi J., Liu X., Liu J., Lyu J., Zhou M., Marszałek K., Trych U. (2020). Effect of high pressure homogenization combined with juice ratio on water-soluble pectin characteristics, functional properties and bioactive compounds in mixed juices. Innov. Food Sci. Emerg. Technol..

[22] Patrignani F., Siroli L., Braschi G., Lanciotti R. (2020). Combined use of natural antimicrobial based nanoemulsions and ultra high pressure homogenization to increase safety and shelf-life of apple juice. Food Control.

[23] Patrignani F., Tabanelli G., Siroli L., Gardini F., Lanciotti R. (2013). Combined effects of high pressure homogenization treatment and citral on microbiological quality of apricot juice. Int. J. Food Microbiol..

[24] Bevilacqua A., Petruzzi L., Perricone M., Speranza B., Campaniello D., Sinigaglia M., Corbo M.R. (2018). Nonthermal technologies for fruit and vegetable juices and beverages: Overview and advances. Compr. Rev. Food Sci. Food Saf..

[25] Garcia C., Guerin M., Souidi K., Remize F. (2020). Lactic fermented fruit or vegetable juices: Past, present and future. Beverages.

[26] Dimitrellou D., Kandylis P., Kokkinomagoulos E., Hatzikamari M., Bekatorou A. (2021). Emmer-Based Beverage Fortified with Fruit Juices. Appl. Sci..

[27] Serrazanetti D.I., Ndagijimana M., Miserocchi C., Perillo L., Guerzoni M.E. (2013). Fermented tofu: Enhancement of keeping quality and sensorial properties. Food Control.

[28] Mauro C.S.I., Guergoletto K.B., Garcia S. (2016). Development of blueberry and carrot juice blend fermented by Lactobacillus reuteri LR92. Beverages.

[29] Riciputi Y., Serrazanetti D.I., Verardo V., Vannini L., Caboni M.F., Lanciotti R. (2016). Effect of fermentation on the content of bioactive compounds in tofu-type products. J. Funct. Foods.

[30] Lo R., Bansal N., Turner M.S. (2018). Characterisation of Lactococcus lactis isolates from herbs, fruits and vegetables for use as biopreservatives against Listeria monocytogenes in cheese. Food Control.

[31] Siroli L., Patrignani F., Serrazanetti D.I., Vannini L., Salvetti E., Torriani S., Gardini F., Lanciotti R. (2016). Use of a nisin-producing Lactococcus lactis strain, combined with natural antimicrobials, to improve the safety and shelf-life of minimally processed sliced apples. Food Microbiol..

[32] Goodburn C., Wallace C.A. (2013). The microbiological efficacy of decontamination methodologies for fresh produce: A review. Food Control.

[33] Purkiewicz A., Ciborska J., Tańska M., Narwojsz A., Starowicz M., Przybyłowicz K.E., Sawicki T. (2020). The impact of the method extraction and different carrot variety on the carotenoid profile, total phenolic content and antioxidant properties of juices. Plants.

[34] Szczepańska J., Skąpska S., Marszałek K. (2021). Continuous High-pressure Cooling-Assisted Homogenization Process for Stabilization of Apple Juice. Food Bioprocess Technol..

[35] Benjamin O., Gamrasni D. (2020). Microbial, nutritional, and organoleptic quality of pomegranate juice following high-pressure homogenization and low-temperature pasteurization. J. Food Sci..

[36] Szczepańska J., Skąpska S., Połaska M., Marszałek K. (2021). High pressure homogenization with a cooling circulating system: The effect on physiochemical and rheological properties, enzymes, and carotenoid profile of carrot juice. Food Chem..

[37] Diels A.M., Michiels C.W. (2006). High-pressure homogenization as a non-thermal technique for the inactivation of microorganisms. Crit. Rev. Microbiol..

[38] Zamora A., Guamis B. (2015). Opportunities for ultra-high-pressure homogenisation (UHPH) for the food industry. Food Eng. Rev..

[39] Pinho C.R., Franchi M.A., Tribst A.A., Cristianini M. (2011). Effect of ultra high pressure homogenization on alkaline phosphatase and lactoperoxidase activity in raw skim milk. Procedia Food Sci..

[40] Floury J., Bellettre J., Legrand J., Desrumaux A. (2004). Analysis of a new type of high pressure homogeniser. A study of the flow pattern. Chem. Eng. Sci..

[41] Donsì F., Esposito L., Lenza E., Senatore B., Ferrari G. (2009). Production of shelf-stable annurca apple juice with pulp by high pressure homogenization. Int. J. Food Eng..

[42] Stannard C. (1997). Development and use of microbiological criteria for foods. Food Sci. Technol. Today.

[43] Food Safety Authority of Ireland (2016). Report on a Total Diet Study Carried out by the Food Safety Authority of Ireland in the Period 2012–2014.

[44] Calligaris S., Foschia M., Bartolomeoli I., Maifreni M., Manzocco L. (2012). Study on the applicability of high-pressure homogenization for the production of banana juices. LWT-Food Sci. Technol..

[45] Tribst A.A.L., Franchi M.A., de Massaguer P.R., Cristianini M. (2011). Quality of mango nectar processed by high-pressure homogenization with optimized heat treatment. J. Food Sci..

[46] Liu X., Liu J., Bi J., Cao F., Ding Y., Peng J. (2019). Effects of high pressure homogenization on physical stability and carotenoid degradation kinetics of carrot beverage during storage. J. Food Eng..

[47] AIJN (2001). Code of Practice for Evaluation of Fruit and Vegetable Juices. https://aijn.eu/en/the-aijn-code-of-practice.

[48] Leroy F., De Vuyst L. (2004). Lactic acid bacteria as functional starter cultures for the food fermentation industry. Trends Food Sci. Technol..

[49] O’sullivan L., Ross R., Hill C. (2002). Potential of bacteriocin-producing lactic acid bacteria for improvements in food safety and quality. Biochimie.

[50] Sarkar P., Bhunia A.K., Yao Y. (2017). Impact of starch-based emulsions on the antibacterial efficacies of nisin and thymol in cantaloupe juice. Food Chem..

[51] Demir N., Acar J., Bahçeci K.S. (2004). Effects of storage on quality of carrot juices produced with lactofermentation and acidification. Eur. Food Res. Technol..

[52] Yu L.J., Rupasinghe H.V. (2012). Effect of acidification on quality and shelf-life of carrot juice. Can. J. Plant Sci..

[53] Filannino P., Cardinali G., Rizzello C.G., Buchin S., De Angelis M., Gobbetti M., Di Cagno R. (2014). Metabolic responses of Lactobacillus plantarum strains during fermentation and storage of vegetable and fruit juices. Appl. Environ. Microbiol..

[54] Di Cagno R., Filannino P., Gobbetti M. (2017). Lactic acid fermentation drives the optimal volatile flavor-aroma profile of pomegranate juice. Int. J. Food Microbiol..

[55] Ricci A., Cirlini M., Levante A., Dall’Asta C., Galaverna G., Lazzi C. (2018). Volatile profile of elderberry juice: Effect of lactic acid fermentation using L. plantarum, L. rhamnosus and L. casei strains. Food Res. Int..

[56] Fukuda T., Tanaka H., Ihori H., Okazaki K., Shinano T., Fukumori Y. (2013). Identification of important volatiles in fresh and processing carrot varieties: Using kuroda and flakee types. Food Sci. Technol. Res..

[57] Dolan L.C., Matulka R.A., Burdock G.A. (2010). Naturally occurring food toxins. Toxins.

[58] Septembre-Malaterre A., Remize F., Poucheret P. (2018). Fruits and vegetables, as a source of nutritional compounds and phytochemicals: Changes in bioactive compounds during lactic fermentation. Food Res. Int..

