# Supplementary material for: High-Pressure Homogenization and Biocontrol Agent as Innovative Approaches Increase Shelf Life and Functionality of Carrot Juice
Source: Foods. 2021 Dec 4;10(12):2998. doi: 10.3390/foods10122998 (PMC8701166; doi:10.3390/foods10122998)
Supplement: Supplementary file 1 [file foods-10-02998-s001.zip › foods-1431192-supplementary.pdf]

Supplementary Materials

Table S1. GC/MS/SPME profiles (expressed as relative abundance) of Carrot juices immediately after production and during their storage at 4 and 10 °C. Samples were treated with high pressure homogenization (HPH, 150MPa x 3 passes) or HPH combined with *L. lactis* LBG2 (L) fermentation. As control (CTRL), a sample that passed through 0.1 MPa was used. Analyses were performed only on samples collected within their shelf-life period (NT, 6 days; HPH, 9 days; HPH+LBG2, 12 days for samples stored at 4 °C or NT, 2 days; HPH, 5 days; HPH+LBG2, 7 days, for samples stored at 10 °C). Results are the mean of 3 biological repetitions (n=3).

				Storage at 4 °C						Storage at 10 °C					
**Time (days)**	**0**			**6**			**9**		**12**	**2**			**5**		**7**
**Molecules**	**CTRL 1**	**HPH**	**HPH+LBG2**	**CTRL 1**	**HPH**	**HPH+LBG2**	**HPH**	**HPH+LBG2**	**HPH+LBG2**	**CTRL**	**HPH**	**HPH+LBG2**	**HPH**	**HPH+LBG2**	**HPH+LBG2**
acetaldehyde	0.1	0.4	0.3	0.5	0.3	0.6	0.3	0.6	0.5	0.5	0.3	0.6	0.1	0.6	0.6
Butanal, 2-methyl-	0.0	0.0	0.0	0.0	0.0	0.0	0.0	0.0	0.0	0.0	0.0	0.0	0.0	0.0	0.0
Butanal, 3-methyl-	0.0	0.0	0.2	0.0	0.0	0.2	0.0	0.2	0.1	0.0	0.1	0.2	0.0	0.2	0.2
Hexanal	0.0	0.4	0.0	0.0	0.0	0.2	0.0	0.2	0.1	0.0	0.0	0.0	0.0	0.2	0.1
Heptanal	0.1	0.0	0.0	0.0	0.0	0.0	0.0	0.0	0.0	0.0	0.0	0.0	0.1	0.0	0.0
Octanal	0.0	0.0	0.0	0.0	0.0	0.0	0.0	0.0	0.1	0.0	0.0	0.0	0.5	0.0	0.0
nonanal	0.0	0.0	0.0	0.1	0.2	0.2	0.2	0.2	0.2	0.0	0.1	0.2	0.0	0.2	0.3
furfural	0.0	0.0	0.0	0.0	0.1	0.0	0.1	0.0	0.0	0.0	0.0	0.0	0.0	0.0	0.0
decanal	0.0	0.0	0.0	0.1	0.2	0.1	0.2	0.0	0.0	0.0	0.0	0.0	0.0	0.1	0.1
2 nonenal	0.1	0.1	0.0	0.1	0.0	0.2	0.0	0.1	0.0	0.1	0.1	0.0	0.1	0.0	0.1
benzeneacetaldehyde	0.0	0.1	0.3	0.1	0.1	0.2	0.1	0.2	0.2	0.2	0.1	0.3	0.0	0.2	0.2
4 ethyl benzaldehyde	0.0	0.0	0.0	0.0	0.0	0.1	0.0	0.1	0.1	0.0	0.0	0.0	0.0	0.1	0.1
**Aldehydes**	**0.4**	**1.0**	**0.9**	**0.9**	**0.9**	**1.7**	**1.0**	**1.5**	**1.3**	**0.8**	**0.7**	**1.3**	**0.8**	**1.4**	**1.7**
Diacetyl	0.0	0.0	0.4	0.0	0.0	0.4	0.0	0.4	0.5	0.0	0.0	0.5	0.0	0.4	0.4
Methyl Isobutyl Ket	1.7	0.9	2.1	1.0	0.4	0.7	0.2	0.7	0.6	1.7	1.3	1.0	0.6	0.9	0.5
5-methyl-3-Hexanone	0.0	0.3	0.3	0.2	0.1	0.2	0.2	0.2	0.0	0.3	0.3	0.0	0.0	0.3	0.0
3,4-dimethyl-2-pentanone	0.0	0.0	0.2	0.1	0.0	0.0	0.0	0.0	0.0	0.3	0.5	0.1	0.0	0.0	0.0
4 methyl 2 hexanone	0.6	0.0	0.5	0.0	0.0	2.5	0.0	0.0	0.2	0.9	0.0	0.3	0.0	0.0	0.0
4-methyl-3-Penten-2-one	1.2	0.7	1.1	0.7	0.2	0.4	0.3	0.5	0.5	0.8	0.7	0.7	0.0	0.6	0.3
2,6-dimethyl-4-heptanone	7.3	3.4	9.8	3.2	0.8	1.5	0.8	1.7	1.8	6.6	3.7	3.8	0.0	2.6	1.4
2,2,3 trimethyl-cyclobutanone	0.0	0.0	0.0	0.0	0.0	0.0	0.1	0.0	0.3	0.0	0.0	0.0	0.0	0.0	0.0
2,4 pentadione	0.0	0.0	0.1	0.0	0.0	0.0	0.0	0.0	0.0	0.0	0.0	0.0	0.0	0.0	0.0
4,6-dimethyl-2-heptanone	0.0	0.1	0.2	0.0	0.0	0.2	0.4	0.0	0.3	0.1	0.1	0.0	0.0	0.0	0.0
Acetoin	0.0	0.0	0.0	0.0	0.0	0.0	0.1	0.0	0.1	0.0	0.0	0.0	0.0	0.0	0.0
1-hydroxy-2-propanone	0.0	0.0	0.0	0.0	0.1	0.0	0.0	0.0	0.0	0.0	0.0	0.0	0.0	0.0	0.0
6-methyl-5-Hepten-2-one	0.2	0.1	0.1	0.0	0.0	0.1	0.1	0.1	0.2	0.0	0.2	0.2	0.1	0.1	0.1
2-nonanone	0.0	0.1	0.1	0.1	0.2	0.1	0.3	0.2	0.2	0.1	0.1	0.0	0.1	0.1	0.2
Acetophenone	0.0	0.1	0.0	0.0	0.1	0.1	0.1	0.0	0.1	0.1	0.1	0.0	0.0	0.0	0.1
**Ketones**	**11.0**	**5.5**	**14.8**	**5.4**	**2.0**	**6.1**	**2.4**	**3.8**	**4.8**	**11.0**	**6.8**	**6.6**	**0.8**	**4.9**	**2.9**
Ethanol	0.1	0.1	0.1	0.5	0.4	0.2	0.4	0.2	0.1	0.6	0.1	0.3	0.1	0.2	0.2

3-methyl-1-butanol	0.0	0.0	0.1	0.1	0.1	0.4	0.0	0.4	0.0	0.1	0.0	0.0	0.0	0.4	0.5
2 hexanol	0.2	0.0	0.0	0.0	0.0	0.0	0.0	0.0	0.0	0.0	0.0	0.1	0.0	0.0	0.0
3 Heptanol	0.0	0.0	0.0	0.1	0.0	0.0	0.0	0.0	0.0	0.0	0.0	0.1	0.1	0.0	0.0
1-hexanol	0.0	0.0	0.0	0.5	0.9	0.0	0.1	0.0	0.0	0.1	0.0	0.0	0.0	0.1	0.1
1 octen 3ol	0.0	0.0	0.0	0.0	0.1	0.0	0.1	0.0	0.0	0.0	0.0	0.1	0.1	0.0	0.0
1-heptanol	0.0	0.0	0.0	0.2	0.2	0.0	0.2	0.0	0.0	0.1	0.0	0.2	0.0	0.1	0.0
2-ethyl-1-hexanol	0.0	0.1	0.1	0.2	0.2	0.2	0.2	0.2	0.2	0.1	0.1	0.1	0.3	0.2	0.2
4 heptanol 2,6 dimethyl	0.1	0.1	0.1	0.0	0.1	0.2	0.1	0.2	0.3	0.1	0.0	0.4	0.1	0.2	0.3
1-octanol	0.2	0.2	0.5	0.7	0.5	0.4	0.5	0.4	0.5	0.6	0.1	0.4	0.1	0.4	0.4
Terpinen-4-ol	0.0	0.1	1.1	0.0	0.0	2.6	0.2	3.4	3.8	0.0	0.1	2.6	0.2	2.5	3.7
p-Cymen-8-ol	0.0	0.1	0.0	0.1	0.2	0.1	0.2	0.1	0.1	0.0	0.1	0.1	0.0	0.1	0.1
**Alcohols**	**0.7**	**0.7**	**2.1**	**2.3**	**2.6**	**4.1**	**1.9**	**4.9**	**5.0**	**1.7**	**0.6**	**4.3**	**0.9**	**4.1**	**5.5**
Acetic acid	0.0	0.3	0.4	0.0	0.2	0.4	0.2	0.3	0.3	0.1	0.1	0.3	0.0	0.2	0.4
**Acids**	**0.0**	**0.3**	**0.4**	**0.0**	**0.2**	**0.4**	**0.2**	**0.3**	**0.3**	**0.1**	**0.1**	**0.3**	**0.0**	**0.2**	**0.4**
L-bornyl acetate	6.0	2.6	2.7	3.7	2.2	1.6	2.3	2.1	2.1	4.6	2.0	2.4	2.8	2.0	1.9
Geranyl acetate	0.5	0.3	0.2	0.4	0.2	0.3	0.2	0.3	0.3	0.3	0.2	0.3	0.2	0.3	0.3
Geranyl butyrate	3.1	2.2	2.3	3.1	1.8	2.2	1.9	2.0	2.4	2.6	1.7	2.3	2.0	2.5	2.4
4-Methyl-2-pentyl acetate	0.1	0.1	0.1	0.0	0.0	0.0	0.0	0.0	0.0	0.1	0.1	0.0	0.0	0.0	0.0
Neryl acetate	0.1	0.1	0.2	0.1	0.1	0.1	0.0	0.1	0.1	0.1	0.1	0.1	0.0	0.1	0.1
**Esters**	**9.8**	**5.3**	**5.5**	**7.3**	**4.3**	**4.2**	**4.4**	**4.5**	**4.9**	**7.6**	**4.1**	**5.1**	**5.0**	**4.9**	**4.7**
α-pinene	2.0	5.8	3.9	3.6	8.5	4.6	8.6	5.0	4.2	2.7	7.0	3.9	5.7	4.2	4.9
Camphene	0.0	0.2	0.2	0.3	0.2	0.2	0.2	0.2	0.1	0.4	0.2	0.0	0.0	0.1	0.2
β-pinene	0.9	1.0	0.6	1.1	1.3	0.7	1.3	0.9	0.8	1.0	1.3	0.7	5.2	0.7	0.6
β-phellandrene	0.3	4.0	0.1	1.8	4.4	0.0	4.5	2.2	2.3	0.0	5.1	1.9	0.0	2.1	2.4
β-myrcene	0.0	3.6	2.4	0.1	0.1	3.0	0.1	2.1	0.0	2.0	3.6	3.1	0.0	0.1	3.3
α-phellandrene	0.0	0.0	0.0	0.1	0.2	0.1	0.2	0.1	0.3	0.0	0.1	0.4	0.0	0.1	0.0
α-terpinene	0.5	2.8	2.8	0.7	2.4	2.1	2.5	2.4	1.9	0.7	2.6	1.9	1.5	2.0	2.1
D-limonene	2.4	3.0	1.7	2.2	2.4	2.0	2.5	2.3	2.0	2.9	2.9	2.5	2.6	1.9	1.9
β-thujene	0.1	0.5	0.6	0.1	0.4	0.5	0.4	0.5	0.4	0.4	0.5	0.4	0.4	0.4	0.5
Ocimene	0.4	0.5	0.3	0.5	0.3	0.4	0.3	0.4	0.4	0.4	0.5	0.0	0.4	0.3	0.3
γ-Terpinene	13.0	10.2	8.6	11.2	9.6	10.3	10.0	10.2	9.5	11.6	9.9	8.5	10.4	8.8	9.5
ϱ-Cymene	5.6	5.5	4.3	3.7	7.2	2.6	7.4	3.0	2.8	6.3	6.3	2.5	8.0	2.0	2.6
Terpinolene	16.2	15.8	13.8	13.0	16.0	15.3	14.2	16.6	14.3	14.5	16.5	13.5	18.7	13.7	14.1
Isoterpinolene	0.0	0.2	0.2	0.0	0.0	0.1	0.0	0.1	0.0	0.2	0.1	0.4	0.0	0.0	0.1
Allo-Ocimene	0.0	0.1	0.1	0.0	0.0	0.0	0.0	0.0	0.0	0.1	0.0	0.0	0.0	0.0	0.0
(E,Z)-alloocimene	0.1	0.1	0.2	0.0	0.0	0.0	0.0	0.0	0.0	0.2	0.0	0.1	0.1	0.0	0.0
Cosmene	0.1	0.0	0.0	0.0	0.0	0.0	0.0	0.0	0.1	0.0	0.0	0.0	0.1	0.0	0.1
β-Terpineol	0.0	0.1	0.0	0.0	0.0	0.1	0.1	0.1	0.1	0.0	0.1	0.2	0.0	0.1	0.1
p-Cymenene	1.2	1.5	1.5	0.9	1.4	0.9	1.4	1.1	1.0	1.5	1.1	0.9	1.2	0.9	0.9
δ-Elemene	0.3	0.2	0.0	0.4	0.1	0.2	0.1	0.1	0.2	0.2	0.1	0.2	0.0	0.2	0.2
Calarene	0.1	0.1	0.0	0.0	0.0	0.0	0.0	0.0	0.1	0.1	0.0	0.1	0.0	0.0	0.1
Copaene	0.0	0.0	0.0	0.1	0.0	0.0	0.0	0.0	0.0	0.0	0.0	0.0	0.0	0.0	0.0
Camphor	0.0	0.0	0.0	0.0	0.0	0.1	0.0	0.0	0.0	0.1	0.1	0.0	0.0	0.0	0.1
α-Bergamotene	0.6	0.5	0.5	0.8	0.4	0.9	0.4	0.7	0.9	0.6	0.5	0.7	0.3	1.0	0.7
Caryophyllene	21.4	19.8	21.0	25.6	20.4	24.7	21.1	22.4	26.1	19.5	19.7	22.2	26.7	27.6	25.0
α-Farnesene	0.0	0.0	0.0	0.0	0.7	0.0	0.7	1.0	0.0	0.0	0.0	0.9	0.0	1.3	0.0
β-Farnesene	0.5	0.8	0.7	1.0	0.6	1.4	0.6	1.2	1.4	0.4	0.6	1.1	0.7	1.6	1.3
Humulene	0.6	1.2	1.0	0.7	0.4	1.0	0.5	0.8	1.0	0.7	0.6	0.7	1.0	1.0	0.8
α-Caryophyllene	1.8	2.8	1.9	3.2	2.2	3.6	2.2	3.1	3.6	1.8	2.3	2.4	2.8	3.9	3.3
Isoborneol	1.2	0.5	0.7	2.0	0.6	0.6	0.6	0.6	0.6	2.4	0.5	0.6	0.3	0.6	0.6
α-Zingiberene	0.3	0.3	0.2	0.4	0.1	0.5	0.1	0.4	0.4	0.2	0.2	0.4	0.2	0.5	0.4

β-Bisabolene	1.0	1.1	1.0	1.7	0.7	1.7	0.7	1.3	1.6	0.8	0.8	1.3	0.9	1.9	1.5
α-Bergamotene	0.1	0.2	0.1	0.2	0.1	0.2	0.1	0.2	0.2	0.1	0.1	0.3	0.0	0.2	0.2
α-Patchoulene	0.5	0.5	0.7	1.3	1.2	0.9	1.2	0.8	1.0	0.6	0.3	0.6	0.0	1.2	1.1
α-Curcumene	1.8	1.9	2.5	2.1	1.4	1.7	1.5	1.6	1.8	2.1	1.2	1.9	1.9	2.2	1.7
Geraniol	0.2	0.1	0.2	0.6	0.5	0.2	0.6	0.2	0.2	0.5	0.2	0.2	0.3	0.2	0.2
Geranyl acetone	0.1	0.1	0.1	0.2	0.6	0.1	0.6	0.1	0.2	0.2	0.1	0.1	0.2	0.1	0.1
Isocaryophyllene oxide	0.0	0.0	0.0	0.0	0.2	0.0	0.2	0.0	0.0	0.0	0.0	0.0	0.0	0.0	0.0
**Terpenes and Terpenoids**	**73.3**	**84.5**	**71.7**	**79.4**	**84.7**	**80.6**	**85.0**	**81.4**	**79.5**	**75.0**	**84.9**	**74.4**	**89.6**	**80.6**	**80.9**
1,3,8-p-menthatriene	0.0	0.1	0.1	0.1	0.2	0.1	0.2	0.1	0.0	0.1	0.1	0.1	0.0	0.1	0.0
Myristicin	2.4	1.2	0.9	1.7	1.5	1.1	1.5	1.1	1.2	1.8	0.9	1.1	1.1	1.0	1.3
**Others**	**2.4**	**1.3**	**1.0**	**1.8**	**1.6**	**1.1**	**1.7**	**1.1**	**1.2**	**1.9**	**1.0**	**1.1**	**1.1**	**1.1**	**1.3**
**Total area** [3]	**17500**	**32800**	**17600**	**21400**	**22500**	**28000**	**21700**	**26300**	**23000**	**18600**	**26800**	**22400**	**18900**	**22600**	**24400**

Data are the mean of three different samples. The variability coefficient ranged between 5% and 7%. 1. Sample treated at 0.1 MPa. 2. Value equal to 0 are Under detection limit. [3]. Arbitrary units (x100000).
